# Assessment of the load-velocity profile in the free-weight prone bench pull exercise through different velocity variables and regression models

**DOI:** 10.1371/journal.pone.0212085

**Published:** 2019-02-27

**Authors:** Amador García-Ramos, David Ulloa-Díaz, Paola Barboza-González, Ángela Rodríguez-Perea, Darío Martínez-García, Mauricio Quidel-Catrilelbún, Francisco Guede-Rojas, Jesualdo Cuevas-Aburto, Danica Janicijevic, Jonathon Weakley

**Affiliations:** 1 Department of Physical Education and Sport, Faculty of Sport Sciences, University of Granada, Granada, Spain; 2 Department of Sports Sciences and Physical Conditioning, Faculty of Education, CIEDE, Catholic University of Most Holy Concepción, Concepción, Chile; 3 Faculty of Education, Universidad Andres Bello, Concepción, Chile; 4 Faculty of Rehabilitation Sciences, Kinesiology, Universidad Andres Bello, Concepción, Chile; 5 Faculty of Sport and Physical Education, University of Belgrade, The Research Centre, Belgrade, Serbia; 6 Institute for Sport, Physical Activity and Leisure, Leeds Beckett University, Leeds, United Kingdom; University of New Brunswick, CANADA

## Abstract

This aims of this study were (I) to determine the velocity variable and regression model which best fit the load-velocity relationship during the free-weight prone bench pull exercise, (II) to compare the reliability of the velocity attained at each percentage of the one-repetition maximum (1RM) between different velocity variables and regression models, and (III) to compare the within- and between-subject variability of the velocity attained at each %1RM. Eighteen men (14 rowers and four weightlifters) performed an incremental test during the free-weight prone bench pull exercise in two different sessions. General and individual load-velocity relationships were modelled through three velocity variables (mean velocity [MV], mean propulsive velocity [MPV] and peak velocity [PV]) and two regression models (linear and second-order polynomial). The main findings revealed that (I) the general (Pearson's correlation coefficient [*r*] range = 0.964–0.973) and individual (median *r* = 0.986 for MV, 0.989 for MPV, and 0.984 for PV) load-velocity relationships were highly linear, (II) the reliability of the velocity attained at each %1RM did not meaningfully differ between the velocity variables (coefficient of variation [CV] range = 2.55–7.61% for MV, 2.84–7.72% for MPV and 3.50–6.03% for PV) neither between the regression models (CV range = 2.55–7.72% and 2.73–5.25% for the linear and polynomial regressions, respectively), and (III) the within-subject variability of the velocity attained at each %1RM was lower than the between-subject variability for the light-moderate loads. No meaningful differences between the within- and between-subject CVs were observed for the MV of the 1RM trial (6.02% *vs*. 6.60%; CV_ratio_ = 1.10), while the within-subject CV was lower for PV (6.36% *vs*. 7.56%; CV_ratio_ = 1.19). These results suggest that the individual load-MV relationship should be determined with a linear regression model to obtain the most accurate prescription of the relative load during the free-weight prone bench pull exercise.

## Introduction

The use of technology in sport can provide information for optimizing the prescription and monitoring of training programs [1,2]. Furthermore, improved affordability and availability of technology has enabled the development of new training methodologies such as "velocity-based resistance training" [3–5]. Velocity-based resistance training requires the measurement of velocity in real-time and provides at least three important practical applications: (I) load can be adjusted on a daily basis to match the desired intensity (commonly expressed as a percentage of the one-repetition maximum; 1RM) due to the strong relationship between movement velocity and the load lifted [6,7], (II) the volume of the training session (e.g., the number of exercises per session, sets per exercise or repetitions per set) can be prescribed based off the magnitude of velocity loss due to its close relationship with markers of fatigue [8,9], and (III) the administration of real-time velocity feedback improves motivation and enables the maintenance of higher movement velocities during resistance training, which in turn may stimulate long-term training adaptations [10,11]. Despite these encouraging applications, many aspects related to the velocity-based resistance training approach still need to be investigated to facilitate and optimize the application of this novel strength-training methodology.

One of the most commonly investigated application of velocity-based resistance training is the possibility of using movement velocity to determine which %1RM is being lifted [6,7,12–15]. General load-velocity relationship equations that allow for the estimation of the %1RM based off the velocity recorded against a submaximal load were originally proposed for the bench press exercise [6]. This has been completed across a range of resistance training exercises such as the squat, vertical jump, bench press throw, and bench pull [7,12–15]. Two of the most important methodological considerations when proposing load-velocity relationship equations are the velocity variable (e.g., mean velocity [MV], mean propulsive velocity [MPV] or peak velocity [PV]) and the regression model (e.g., first or second-order polynomials) considered [12,16]. Previous studies have suggested that the modelling of the MV through a linear regression should be used to maximize the accuracy of the load-velocity relationship during the bench press exercise [12,16]. However, due to the unique movement patterns of each exercise, it is important to explore whether the previous findings obtained with the bench press exercise could be extended to another commonly used upper-body exercise such as the prone bench pull.

Two studies have been conducted to explore the general load-velocity relationship during the prone bench pull exercise [14,17]. Sánchez-Medina et al. [14] examined the load-velocity relationship during the bench pull exercise, performed in a Smith machine, through a polynomial regression model using both MV and MPV variables. Furthermore, Loturco et al. [17] modelled the load-velocity relationship during the free-weight prone bench pull exercise through a linear regression model using MV, MPV and PV. Both Sánchez-Medina et al. [14] (*r*^2^ = 0.95–0.96 and standard error of the estimate [SEE] = 5.31–5.90%1RM) and Loturco et al. [17] (*r*^2^ = 0.90–0.91 and SEE = 6.27–6.56%1RM) recommended the use of their general load-velocity relationship equations to estimate the %1RM. However, no previous study has directly compared which regression model (linear or second-order polynomial) provides the most accurate method for determining the load-velocity relationship during the bench pull exercise. In addition, the between-session reliability of the load-velocity relationship (i.e., the velocity attained at each %1RM) has never been evaluated during the bench pull exercise. The load-velocity relationship should be determined in two different days to calculate the within-subject variability of the velocity attained at each %1RM. Note that Sánchez-Medina et al. [14] and Loturco et al. [17] only examined the between-subject variability since their subjects were only tested once. Therefore, we believe that determining the precision and reliability of both regression models (linear and polynomial) to obtain the load-velocity relationship during the bench pull exercise presents practical interest.

To address the existing gaps in the literature, further examination of the load-velocity relationship during the free-weight prone bench pull exercise is required. Therefore, the aims of this study were (I) to determine the regression model (linear *vs*. second-order polynomial) that is able to better fit the load-velocity relationship considering different velocity variables (MV, MPV and PV), (II) to compare the reliability of the velocity attained at each %1RM between different velocity variables and regression models, and (III) to compare the within- and between-subject variability of the velocity attained at each %1RM. We hypothesized that (I) the general and individual load-velocity relationships would be highly linear regardless of the velocity variable considered [14,17], (II) the PV and the linear regression model would provide the lowest within-subject variability of the velocity attained at each %1RM [12,16], and (III) the within-subject variability of the velocity attained at each %1RM would be lower than the between-subject variability [16].

## Materials and methods

### Subjects

Eighteen men (14 rowers and four weightlifters) participated in this study (mean ± standard deviation [SD]: age = 20.6 ± 2.7 years [range: 16–25 years]; body mass = 72.5 ± 9.1 kg; height = 1.74 ± 0.07 m; prone bench pull 1RM = 89.9 ± 12.6 kg; prone bench pull training experience = 6.2 ± 4.2 years). Subjects were not allowed to perform any strenuous exercise during the 24 hours preceding each testing session. None of the subjects had injuries or musculoskeletal pain that could compromise the results of the present study. Prior to testing, subjects were informed about the research purpose and procedures, and they or their legal guardians (for subjects aged < 18) gave written consent to participate in the study. The experiment was approved by the local Ethics Committee of the University of Granada (491/CEIH/2018) according to the Declaration of Helsinki.

### Experimental design

A repeated-measures design was used to investigate different methodological aspects related to the assessment of the load-velocity profile during the free-weight prone bench pull exercise. Subjects performed an incremental loading test from 20 kg until the 1RM load, during two sessions that were separated by 72–96 hours. General and individual load-velocity relationships were modelled through three different velocity variables (MV, MPV and PV) and two regression models (linear and second-order polynomial). All testing sessions were held between 15:00–19:00 hours.

### Testing procedures

Each testing session began with a standardized warm-up consisting of 5 minutes of jogging, followed by joint mobility exercises, and one set of five repetitions performed against an external load of 20 kg (i.e., the mass of the Olympic barbell used during the test) during the free-weight prone bench pull exercise. The initial external load of the incremental loading test was 20 kg for all subjects and it was progressively increased in 10 kg until the MV was lower than 0.80 m·s^-1^ (≈ 70%1RM). Afterwards, the load was increased in steps of 5 to 1 kg until the 1RM load was achieved. Three repetitions were performed with light loads (MV > 1.10 m·s^−1^), two with medium loads (1.10 m·s^−1^ ≤ MV ≤ 0.80 m·s^−1^) and one with heavy loads (MV < 0.80 m·s^−1^). Intra-set rest between repetitions was 10 seconds and inter-set rest was 5 minutes. Subjects received feedback of velocity immediately after each repetition and were encouraged to perform all repetitions at the maximal intended velocity.

The bench pull exercise was performed with a standard Olympic barbell and calibrated weight plates (Eleiko, Halmstad, Sweden). The initial position involved the subjects lying down in a prone position, the chin in contact with the bench, elbows fully extended, and a prone grip of the barbell slightly wider than shoulder width. From that position, subjects were instructed to pull the barbell as fast as possible until it contacted with the underside of the bench. A repetition was not considered valid if the barbell did not contact the underside of the bench. The legs were held during all repetitions by a researcher and the chin remained in contact with the bench at all times. The thickness of the bench was 8.5 cm.

### Measurement equipment and data analysis

A linear velocity transducer (T-Force System; Ergotech, Murcia, Spain) was attached to the right side of the barbell and recorded vertical velocity at a frequency of 1,000 Hz. Three velocity variables were considered in the present study: MV–mean velocity value from the start of the concentric phase until the velocity of the barbell is 0 m·s^-1^; MPV–mean velocity value from the start of the concentric phase until the acceleration of the barbell is lower than gravity (-9.81 m·s^-2^); PV–maximum instantaneous velocity value reached during the concentric phase [12]. Only the repetition with the highest velocity value (MV, MPV or PV was used as the criterion for their respective load-velocity relationships) of each loading condition was used for subsequent analysis.

Repetitions of all subjects were pooled together for determining the general load-velocity relationships (6 equations were provided: 3 variables × 2 regression models). The individual-load velocity relationships were also determined and the velocities attained at each %1RM (in 5% increments from 20%1RM to 100%1RM) were depicted. The general and individual load-velocity relationships were modelled applying both first-order and second-order polynomials to the data [16]. The data of the first testing session were used to explore the general load-velocity relationships, while the data of both testing sessions were used to analyze the individual load-velocity relationships.

### Statistical analyses

Descriptive data are presented as means and SD, while the Pearson's correlation coefficient (*r*) is presented through their median values and range. The relationship between relative load (%1RM) and the three velocity variables (MV, MPV and PV) was assessed by means of linear and second-order polynomial regression models. The goodness of fit of the general and individual load-velocity relationships was assessed by the *r* coefficient and the *F* statistic. The linearity of the individual load-velocity relationships was also compared between the three velocity variables through a one-way repeated measures ANOVA applied on the *r* coefficients. The standard error of the measurement (within-subject SD), the coefficient of variation (CV (%) = $\frac{Within-subject\;SD}{Subjects\prime \;mean\;score}\times 100$) and the intraclass correlation coefficient (ICC, model 3.1) were used to explore reliability of the velocity values attained at each %1RM obtained from the individual load-velocity relationships as well as the velocity of the 1RM trial. Acceptable reliability was determined as a CV < 10% [18]. The ratio between two CVs was used to compare the reliability between the different velocity variables and regression models. The CV ratio was also used to compare the within-subject CV of the velocity values attained at each %1RM against the between-subject CV (CV (%) = $\frac{Between-subject\;SD}{Subjects\prime \;mean\;score}\times 100$). The value of 1.15 was considered as the smallest important CV ratio [19]. The reliability analysis was performed by means of a custom spreadsheet [20], whereas SPSS (version 22.0; SPSS, Inc., Chicago, IL) was used for other statistical analyses. Alpha was set at 0.05.

## Results

**Fig 1:**
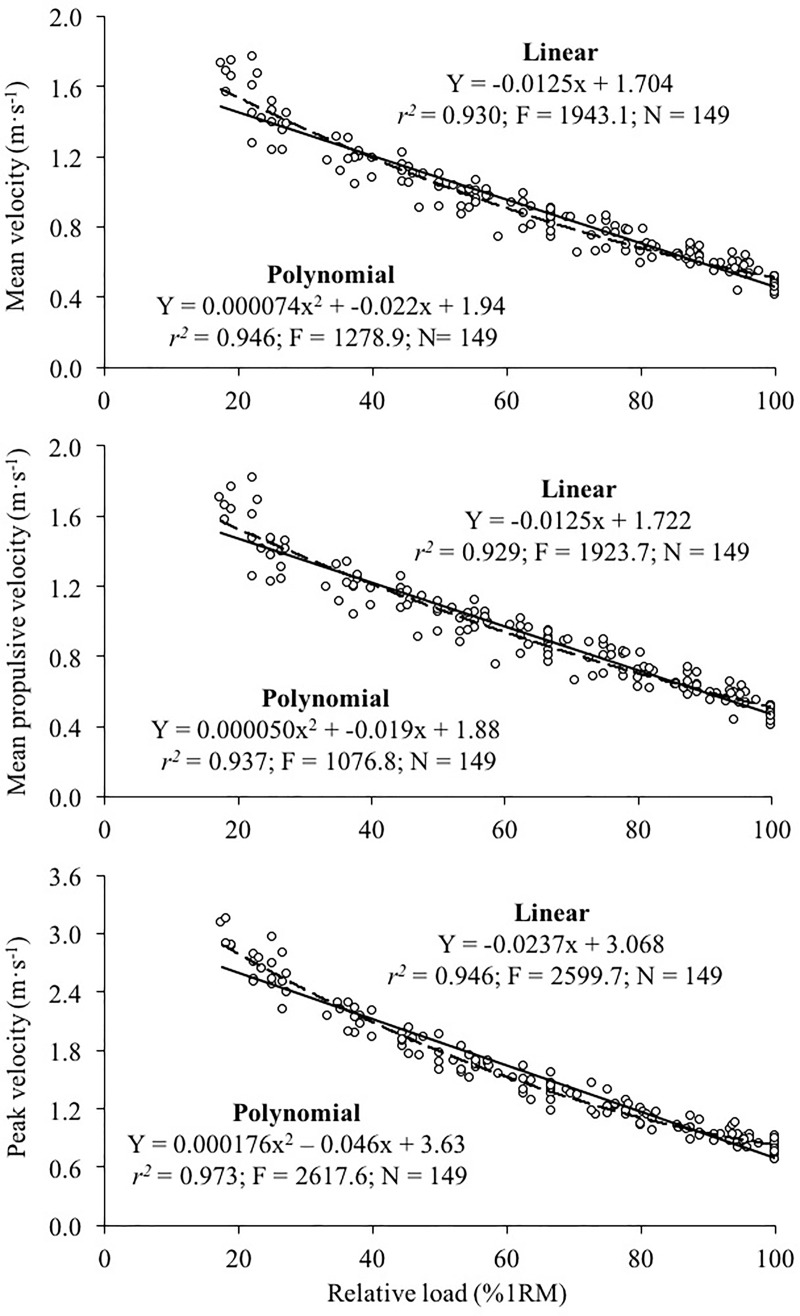
**Generalized across the subjects relationship between the relative load (%1RM) and mean velocity (upper panel), mean propulsive velocity (middle panel) and peak velocity (lower panel) in the bench pull exercise.** The linear (solid line) and second-order polynomial (dashed line) regression equations are depicted. *r*^*2*^, Pearson's coefficient of determination; F, F statistic; N = number of trials included in the regression analysis.

The general load-velocity relationships were strong for the three velocity variables (*r* ≥ 0.93; Fig 1). The linear regression model provided a higher *F* statistic compared to the polynomial regression model for MV and MPV, while no meaningful differences in the magnitude of the *F* statistic was observed between both regression models for PV. The individual load-velocity relationships were also highly linear for the three velocity variables (median *r* [range] = 0.986 [0.945, 0.999] for MV, 0.989 [0.939, 0.998] for MPV, and 0.984 [0.967, 0.997] for PV). The linearity of the individual load-velocity relationships did not significantly differ between the three velocity variables (F = 1.26, *p* = 0.296). The average and between-subject SD of the velocity values associated with each %1RM are depicted in Table 1.

**Table 1:** Velocity values associated with each relative load (%1RM) obtained from the individual load-velocity relationships modelled through linear and second-order polynomial regression models.

Load (%1RM)	Mean velocity (m·s^-1^)		Mean propulsive velocity (m·s^-1^)		Peak velocity (m·s^-1^)	
	Linear	Polynomial	Linear	Polynomial	Linear	Polynomial
20	1.45 ± 0.13	1.53 ± 0.14	1.47 ± 0.14	1.52 ± 0.15	2.58 ± 0.16	2.77 ± 0.17
25	1.39 ± 0.12	1.44 ± 0.12	1.41 ± 0.13	1.44 ± 0.13	2.46 ± 0.15	2.59 ± 0.15
30	1.33 ± 0.11	1.35 ± 0.11	1.34 ± 0.12	1.36 ± 0.12	2.34 ± 0.14	2.41 ± 0.14
35	1.26 ± 0.10	1.27 ± 0.10	1.28 ± 0.11	1.29 ± 0.11	2.23 ± 0.13	2.24 ± 0.13
40	1.20 ± 0.09	1.19 ± 0.09	1.22 ± 0.10	1.21 ± 0.10	2.11 ± 0.12	2.08 ± 0.12
45	1.14 ± 0.09	1.12 ± 0.08	1.16 ± 0.09	1.14 ± 0.09	1.99 ± 0.11	1.93 ± 0.11
50	1.08 ± 0.08	1.04 ± 0.08	1.09 ± 0.08	1.07 ± 0.09	1.87 ± 0.11	1.79 ± 0.11
55	1.02 ± 0.07	0.97 ± 0.08	1.03 ± 0.08	1.01 ± 0.08	1.76 ± 0.10	1.65 ± 0.10
60	0.95 ± 0.06	0.91 ± 0.07	0.97 ± 0.07	0.94 ± 0.08	1.64 ± 0.09	1.53 ± 0.10
65	0.89 ± 0.06	0.85 ± 0.07	0.91 ± 0.06	0.88 ± 0.07	1.52 ± 0.08	1.41 ± 0.09
70	0.83 ± 0.05	0.79± 0.06	0.84 ± 0.05	0.82 ± 0.07	1.40 ± 0.08	1.30 ± 0.08
75	0.77 ± 0.04	0.73 ± 0.06	0.78 ± 0.05	0.76 ± 0.06	1.29 ± 0.07	1.20 ± 0.08
80	0.70 ± 0.04	0.68 ± 0.05	0.72 ± 0.04	0.70 ± 0.05	1.17 ± 0.07	1.11 ± 0.07
85	0.64 ± 0.04	0.63 ± 0.04	0.66 ± 0.04	0.65 ± 0.04	1.05 ± 0.07	1.02 ± 0.06
90	0.58 ± 0.04	0.59 ± 0.03	0.59 ± 0.04	0.60 ± 0.03	0.93 ± 0.06	0.95 ± 0.05
95	0.52 ± 0.04	0.55 ± 0.03	0.53 ± 0.04	0.55 ± 0.03	0.82 ± 0.07	0.88 ± 0.05
100	0.46 ± 0.04	0.51 ± 0.03	0.47 ± 0.04	0.50 ± 0.03	0.70 ± 0.07	0.83 ± 0.06

Mean ± standard deviation. 1RM, one-repetition maximum

An acceptable reliability (CV < 10%) was observed at all relative loads for the three velocity variables and two regression models (Table 2). A significant ICC value was also observed at all relative loads with the exception of the heavy loads (85–100%1RM) for MV and MPV and the moderate loads (45–60%1RM) for the PV modelled through a polynomial regression model (Table 3). The reliability of the full load-velocity relationships did not meaningfully differ between the velocity variables or between the regression models. However, the MV generally provided the highest reliability with the light loads and the PV was the most reliable variable at higher loads. The linear regression model generally provided a higher reliability (lower CV and higher ICC) at light-moderate relative loads (20–80%1RM), while the polynomial regression model provided a higher reliability with the heavy relative loads (90–100%1RM).

**Table 2:** Within-subject coefficient of variation (CV) with 95% confidence intervals obtained at each relative load for each velocity variable and regression model.

Load (%1RM)	Mean velocity		Mean propulsive velocity		Peak velocity	
	Linear	Polynomial	Linear	Polynomial	Linear	Polynomial
20	2.85 (2.14, 4.27)*,*b*,*c*	3.73 (2.80, 5.59)*b*	3.45 (2.59, 5.18)*	4.79 (3.59, 7.18)	3.62 (2.71, 5.42)	3.82 (2.87, 5.73)*b*
25	2.77 (2.08, 4.16)*,*b*,*c*	3.25 (2.44, 4.87)*b*	3.35 (2.51, 5.02)*	4.13 (3.10, 6.19)	3.59 (2.70, 5.39)	3.67 (2.76, 5.50)
30	2.71 (2.03, 4.06)*b*,*c*	2.90 (2.17, 4.34)*b*,*c*	3.24 (2.43, 4.86)	3.60 (2.70, 5.40)	3.57 (2.68, 5.35)	3.60 (2.70, 5.40)
35	2.64 (1.98, 3.96)*b*,*c*	2.73 (2.05, 4.09)*b*,*c*	3.14 (2.35, 4.70)	3.25 (2.44, 4.87)	3.55 (2.66, 5.32)	3.62 (2.72, 5.43)
40	2.59 (1.95, 3.89)*b*,*c*	2.76 (2.07, 4.14)*c*	3.04 (2.28, 4.55)*c*	3.12 (2.34, 4.67)*c*	3.53 (2.65, 5.29)	3.72 (2.79, 5.58)
45	2.56 (1.92, 3.84)*,*b*,*c*	2.98 (2.23, 4.46)*c*	2.95 (2.21, 4.42)*c*	3.20 (2.40, 4.80)*c*	3.51 (2.63, 5.26)	3.87 (2.91, 5.81)
50	2.55 (1.91, 3.82)*,*c*	3.31 (2.49, 4.97)*c*	2.88 (2.16, 4.31)*,*c*	3.46 (2.60, 5.19)*c*	3.50 (2.63, 5.24)*	4.06 (3.05, 6.09)
55	2.58 (1.93, 3.86)*,*c*	3.71 (2.78, 5.56)	2.84 (2.13, 4.25)*,*c*	3.82 (2.86, 5.72)	3.50 (2.62, 5.24)*	4.26 (3.19, 6.38)
60	2.65 (1.99, 3.98)*,*c*	4.10 (3.08, 6.15)	2.84 (2.13, 4.26)*,*c*	4.20 (3.15, 6.30)	3.51 (2.63, 5.26)*	4.42 (3.32, 6.63)
65	2.79 (2.10, 4.19)*,*c*	4.46 (3.35, 6.69)	2.91 (2.18, 4.36)*,*c*	4.56 (3.42, 6.83)	3.54 (2.66, 5.31)*	4.53 (3.40, 6.79)
70	3.02 (2.26, 4.52)*,*c*	4.74 (3.56, 7.11)	3.07 (2.30, 4.60)*,*c*	4.84 (3.63, 7.26)	3.60 (2.70, 5.40)*	4.55 (3.41, 6.81)
75	3.34 (2.51, 5.01)*	4.92 (3.69, 7.38)	3.35 (2.51, 5.02)*	5.02 (3.77, 7.53)	3.70 (2.78, 5.55)*	4.44 (3.33, 6.65)
80	3.79 (2.84, 5.68)*	4.98 (3.73, 7.46)	3.77 (2.83, 5.65)*	5.07 (3.80, 7.60)	3.87 (2.90, 5.80)	4.20 (3.15, 6.29)*a*,*b*
85	4.39 (3.30, 6.58)	4.91 (3.68, 7.35)	4.36 (3.27, 6.54)	4.98 (3.74, 7.47)	4.13 (3.10, 6.19)	3.84 (2.88, 5.76)*a*,*b*
90	5.19 (3.89, 7.77)	4.76 (3.57, 7.14)	5.18 (3.88, 7.76)	4.82 (3.62, 7.23)	4.52 (3.39, 6.78)	3.53 (2.65, 5.28)*,*a*,*b*
95	6.23 (4.67, 9.34)	4.70 (3.53, 7.05)*	6.26 (4.70, 9.39)	4.77 (3.58, 7.15)*	5.12 (3.84, 7.68)*a*,*b*	3.62 (2.71, 5.42)*,*a*,*b*
100	7.61 (5.71, 11.41)	5.05 (3.79, 7.57)*	7.72 (5.79, 11.57)	5.25 (3.94, 7.87)*	6.03 (4.52, 9.04)*a*,*b*	4.59 (3.44, 6.88)*
All	3.54 ± 1.49	4.00 ± 0.87	3.79 ± 1.37	4.29 ± 0.74	3.91 ± 0.70	4.02 ± 0.38

1RM, one-repetition maximum; All, CV value obtained from the full load-velocity relationship (mean ± standard deviation).

*, significantly more reliable than the other regression model

*a*, significantly more reliable than mean velocity

*b*, significantly more reliable than mean propulsive velocity

*c*, significantly more reliable than peak velocity. Significant differences in reliability were defined as a CV ratio > 1.15.

**Table 3:** Intraclass correlation coefficients (ICC) with 95% confidence intervals obtained at each relative load for each velocity variable and regression model.

Load (%1RM)	Mean velocity		Mean propulsive velocity		Peak velocity	
	Linear	Polynomial	Linear	Polynomial	Linear	Polynomial
20	0.89 (0.72, 0.96)	0.81 (0.57, 0.93)	0.85 (0.65, 0.94)	0.75 (0.44, 0.90)	0.53 (0.10, 0.80)	0.50 (0.06, 0.78)
25	0.89 (0.73, 0.96)	0.84 (0.63, 0.94)	0.86 (0.66, 0.94)	0.79 (0.52, 0.92)	0.53 (0.10, 0.79)	0.48 (0.04, 0.77)
30	0.89 (0.73, 0.96)	0.87 (0.68, 0.95)	0.86 (0.66, 0.94)	0.82 (0.59, 0.93)	0.52 (0.09, 0.79)	0.46 (0.01, 0.76)
35	0.89 (0.73, 0.96)	0.87 (0.69, 0.95)	0.86 (0.67, 0.95)	0.85 (0.63, 0.94)	0.52 (0.08, 0.79)	0.44 (-0.01, 0.75)
40	0.89 (0.72, 0.96)	0.86 (0.67, 0.95)	0.86 (0.67, 0.95)	0.85 (0.64, 0.94)	0.51 (0.07, 0.78)	0.43 (-0.03, 0.74)
45	0.88 (0.71, 0.95)	0.83 (0.61, 0.93)	0.86 (0.67, 0.95)	0.83 (0.60, 0.93)	0.50 (0.06, 0.78)	0.42 (-0.04, 0.73)
50	0.87 (0.70, 0.95)	0.79 (0.52, 0.92)	0.86 (0.66, 0.94)	0.80 (0.53, 0.92)	0.50 (0.05, 0.78)	0.42 (-0.05, 0.73)
55	0.86 (0.67, 0.95)	0.73 (0.42, 0.89)	0.85 (0.65, 0.94)	0.74 (0.44, 0.90)	0.49 (0.05, 0.77)	0.42 (-0.04, 0.74)
60	0.84 (0.63, 0.94)	0.67 (0.31, 0.86)	0.84 (0.62, 0.94)	0.68 (0.33, 0.87)	0.49 (0.05, 0.77)	0.44 (-0.02, 0.74)
65	0.81 (0.56, 0.92)	0.61 (0.21, 0.83)	0.81 (0.57, 0.93)	0.62 (0.23, 0.84)	0.49 (0.05, 0.78)	0.46 (0.00, 0.76)
70	0.76 (0.46, 0.90)	0.54 (0.12, 0.80)	0.77 (0.49, 0.91)	0.56 (0.13, 0.81)	0.50 (0.06, 0.78)	0.48 (0.03, 0.77)
75	0.68 (0.33, 0.87)	0.49 (0.04, 0.77)	0.71 (0.37, 0.88)	0.49 (0.05, 0.78)	0.52 (0.08, 0.79)	0.52 (0.08, 0.79)
80	0.58 (0.16, 0.82)	0.43 (-0.03, 0.74)	0.61 (0.21, 0.83)	0.44 (-0.02, 0.75)	0.54 (0.11, 0.80)	0.56 (0.14, 0.81)
85	0.44 (-0.02, 0.75)	0.38 (-0.10, 0.71)	0.47 (0.02, 0.76)	0.39 (-0.07, 0.72)	0.56 (0.15, 0.81)	0.61 (0.21, 0.83)
90	0.31 (-0.17, 0.67)	0.33 (-0.15, 0.68)	0.33 (-0.15, 0.68)	0.37 (-0.10, 0.71)	0.60 (0.19, 0.83)	0.66 (0.29, 0.86)
95	0.22 (-0.26, 0.61)	0.29 (-0.20, 0.66)	0.22 (-0.26, 0.62)	0.37 (-0.10, 0.71)	0.63 (0.24, 0.84)	0.66 (0.30, 0.86)
100	0.19 (-0.29, 0.59)	0.27 (-0.21, 0.65)	0.18 (-0.30, 0.59)	0.40 (-0.07, 0.72)	0.65 (0.28, 0.85)	0.59 (0.19, 0.83)
All	0.70 (0.25)	0.62 (0.22)	0.69 (0.24)	0.63 (0.19)	0.53 (0.05)	0.50 (0.08)

1RM, one-repetition maximum; All, ICC value obtained from the full load-velocity relationship (mean ± standard deviation).

**Fig 2:**
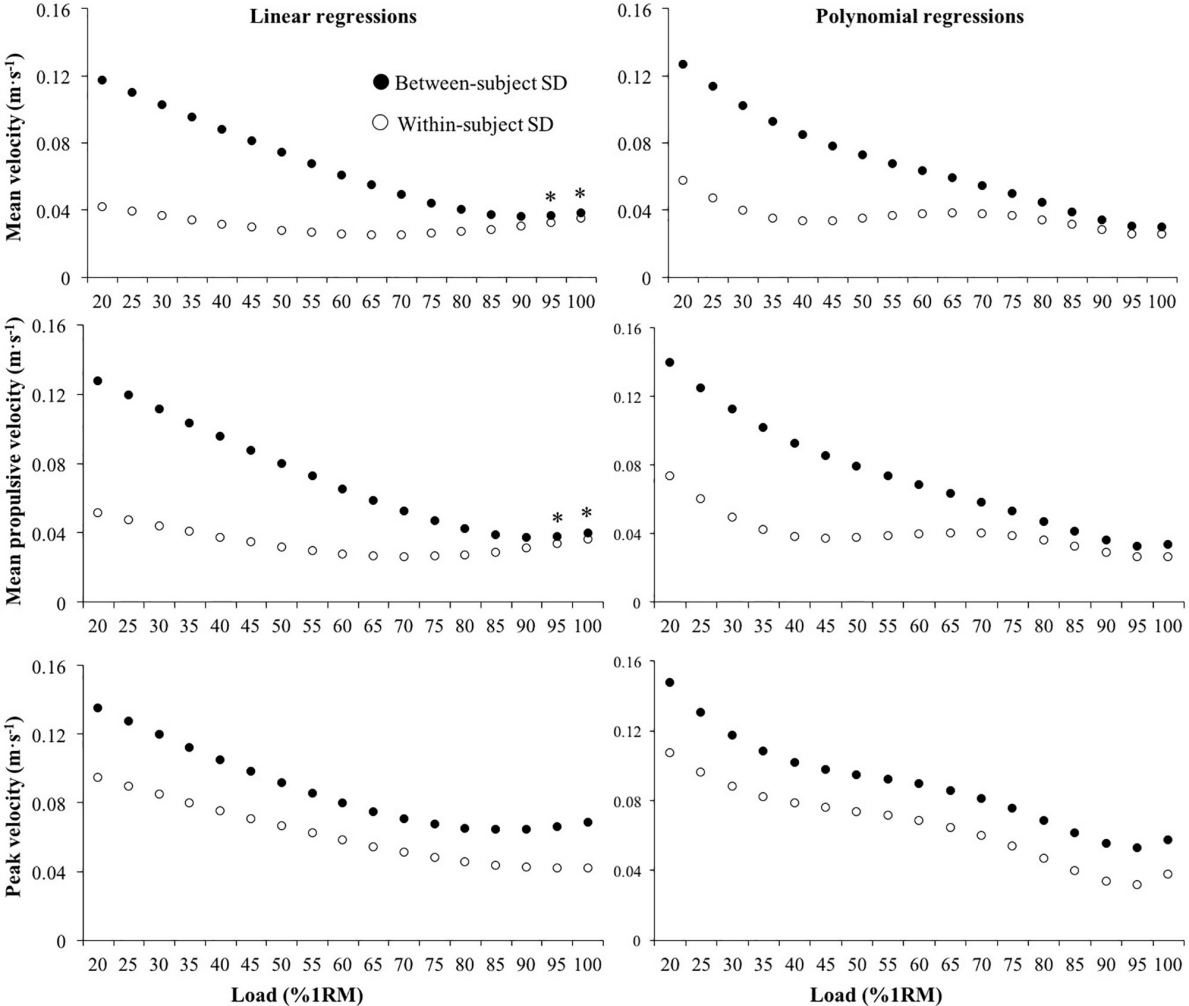
**Within-subject (empty circle) and between-subject (filled circle) standard deviation (SD) of the mean velocity (upper panels), mean propulsive velocity (middle panels) and peak velocity (lower panels) attained at each percentage of the one-repetition maximum (%1RM) obtained from linear (left panels) and second-order polynomial (right panels) regression models.** *, CV ratio between within- and between-subject CVs < 1.1.

The within-subject CV of the velocity values associated with each %1RM were generally lower than the between-subject CV (Fig 2). The magnitude of the differences between the within- and between-subject CV were consistent across the loads for PV, while the differences tended to decrease with the increment of the load for MV and MPV. The MV and PV of the 1RM trial were 0.479 ± 0.032 m·s^-1^ and 0.790 ± 0.060 m·s^-1^, respectively. No meaningful differences between the within- and between-subject CVs were observed for the MV of the 1RM trial (6.02% *vs*. 6.60%; CV ratio = 1.10), while the within-subject CV was lower for PV (6.36% *vs*. 7.56%; CV ratio = 1.19). A low ICC was observed for the MV and PV recorded during the 1RM trial (ICC = 0.18 and 0.31, respectively)

## Discussion

This study was designed to identify the most appropriate velocity variable and regression model to determine the load-velocity relationship during the free-weight prone bench pull exercise, as well as to compare the between- and within-subject variability of the velocity values attained at each %1RM. The main findings of the study revealed that (I) the general and individual load-velocity relationships were highly linear, (II) the reliability of the velocity attained at each %1RM did not systematically differ between the velocity variables or between the regression models, and (III) the within-subject variability of the velocity attained at each %1RM was lower than the between-subject variability at all submaximal loads (%1RM).

Two studies have previously examined the load-velocity relationship during the prone bench pull exercise performed in a Smith machine [14] or with free-weights [17]. Both Sánchez-Medina et al. [14] using a polynomial regression model and two velocity variables (MV and MPV) (*r*^2^ = 0.95–0.96 and standard error of the estimate [SEE] = 5.31–5.90%1RM) and Loturco et al. [17] using a linear regression model and three velocity variables (MV, MPV and PV) (*r*^2^ = 0.90–0.91 and SEE = 6.27–6.56%1RM) recommended the use of their general load-velocity relationship equations to estimate the %1RM. Consequently, it is evident from the results of the current and previous studies that the general load-velocity relationship could be obtained with similar accuracy from the three different velocity variables (MV, MPV and PV) [12,14,17]. In addition, based on the results of the present study, it seems that the modelling of the general load-velocity relationship during the free-weight bench pull exercise through a polynomial regression model does not substantially enhance the accuracy in the estimation of the %1RM compared to the use of a linear regression model. However, it is important to note that due to the limitations of the general load-velocity relationship equations (for details, see García-Ramos and Jaric [21]), the individual load-velocity relationship is recommended for a more accurate prescription of the relative load (%1RM) [7,22,23].

In support of our first hypothesis, the linearity of the individual load-velocity relationships was very high and did not significantly differ between the three velocity variables. This outcome slightly differs from the results previously reported by García-Ramos et al. [12] during the bench press throw exercise in which the MV showed the strongest linearity (*r*^2^ = 0.99), followed by MPV (*r*^2^ = 0.98), and finally PV (*r*^2^ = 0.97). The high linearity of the individual load-velocity relationship observed in the present study, which is in line with the results reported for other exercises [7,12,13,23–25], provides additional evidence regarding the suitability of the linear regression model for assessing the individual load-velocity relationship during basic multi-joint resistance training exercises. However, it should be noted that at very light loads (< 40%1RM) neither the linear nor the polynomial regression model were able to fit with accuracy the recorded velocity values. This is an indicator of the poor ability of general load-velocity relationship equations to estimate low relative loads. This statement could be reinforced by the increasing differences observed between the between-subject and within-subject variability in the velocity values when the relative load is reduced (see Fig 2). Therefore, it is important to consider that the accuracy of general load-velocity relationship equations to estimate the relative load may be compromised even further at high movement velocities.

One of the novelties of the present study is that we also compared the reliability of the velocity values attained at each %1RM between the different individual load-velocity relationships. To our knowledge, this type of comparison has only been performed for the bench press exercise in which the PV and the linear regression model showed the highest reliability [12,16]. However, rejecting our second hypothesis, no systematic differences in the reliability of the individual load-velocity relationship were observed between the velocity variables or between the regression models. Therefore, since the between-subject variability of the velocity recorded at the 1RM trial was lower for the MV compared to the PV, it is reasonable to propose the MV as the most appropriate velocity variable. It should be noted that when heavy loads (> 80%1RM) are lifted there ae no differences between MV and MPV values [26]. In the present study the velocity associated with each %1RM was always similar for the MV and MPV because the barbell is not intentionally braked during the bench pull exercise, as is inevitably the case during the bench press exercise performed against light loads [14,26]. Additionally, it should also be noted that the MV, but not MPV and PV, is a variable reported by all commercial measurement devices that have been designed to measure movement velocity during resistance training. This is accompanied by greater agreement across commercial devices in MV when compared to PV values [27,28].

The third hypothesis of this study was also confirmed, with the within-subject variability of the velocity attained at each %1RM generally being lower than the between-subject variability. In addition, the significant ICC values of the velocity attained at light-moderate (20–80% of 1RM) relative loads provides additional support for the use of individual load-velocity relationships over general load-velocity relationships. In line with results reported for the bench press [16], increases in the relative load were associated with reductions in the differences between the between- and within-subject variability, as well as with lower ICC values. These results indicate that the velocity associated with each %1RM is subject-specific, while the between-subject differences are accentuated at lower relative loads. Interestingly, a non-significant ICC and trivial difference between the between- and within-subject variability were observed for the MV recorded during the 1RM trial. This result presents important practical applications for practitioners, as they could use the average MV attained during the 1RM trials (i.e., 0.48 m·s^-1^) and, using the MV recorded against several submaximal loads, predict the free-weight prone bench pull 1RM. The MV of at least two submaximal loads should be modelled through a linear regression and the 1RM would be estimated as the load associated with a MV of 0.48 m·s^-1^ [29]. Future studies should endeavor to compare the precision of the general and individual load-velocity relationships to predict the 1RM during different resistance training exercises, as well as determine the optimal combinations of submaximal loads that should be considered to maximize the accuracy in the estimation of the 1RM.

In conclusion, the results of the present study support the use of MV and a linear regression model for reliable and accurate determination of the load-velocity profile during the free-weight prone bench pull exercise. Regardless of the velocity variable and regression model considered, the between-subject variability of the velocity attained at the light-moderate loads was always considerably larger than the within-subject variability, suggesting that the individual load-velocity relationship should be considered for a more accurate prescription of the relative load. On the other hand, the low ICC and the similar within- and between-subject variability of the MV attained during the 1RM trial suggest that a standard MV of the 1RM (0.48 m·s^-1^) should be considered for predicting the 1RM load from the MV recorded under several submaximal loads.

## Supporting information

S1 Database Mean velocity (MV) mean propulsive velocity (MPV) and maximum velocity (VMAX) values used to determine the load-velocity relationships. The absolute (kg) and relative (%1RM) loads are indicated.
(XLSM)

## Abbreviations

MV: mean velocity
MPV: mean propulsive velocity
PV: peak velocity
SD: standard deviation
CV: coefficient of variation
ICC: interclass correlation coefficient
r: Pearson's correlation coefficient
SEE: standard error of estimate
1RM: one-repetition maximum
